# Anxiety and Perception of Disease Control in Multiple Sclerosis Subjects Treated with Natalizumab

**DOI:** 10.3390/jcm13010013

**Published:** 2023-12-19

**Authors:** Francesco Corallo, Edoardo Sessa, Carmela Rifici, Maria Cristina De Cola, Marcella Di Cara, Davide Cardile, Giuseppe Venuti, Noemi Bonfiglio, Giangaetano D’Aleo, Angelo Quartarone, Viviana Lo Buono

**Affiliations:** IRCCS Centro Neurolesi Bonino-Pulejo, S.S. 113 Via Palermo, C.da Casazza, 98124 Messina, Italy; francesco.corallo@irccsme.it (F.C.); carmela.rifici@irccsme.it (C.R.); marcella.dicara@irccsme.it (M.D.C.); davide.cardile@irccsme.it (D.C.); giuseppe.venuti@irccsme.it (G.V.); noemi.bonfiglio@gmail.com (N.B.); giangaetano.daleo@irccsme.it (G.D.); angelo.quartarone@irccsme.it (A.Q.); viviana.lobuono@irccsme.it (V.L.B.)

**Keywords:** anxiety, disease control, multiple sclerosis, natalizumab, progressive multifocal leukoencephalopathy

## Abstract

Multiple sclerosis subjects treated with natalizumab face anxiety about developing progressive multifocal leukoencephalopathy (PML), besides the psychological distress caused by the disease. The aim of this study is to investigate whether increasing the frequency of neurological and nuclear magnetic resonance screening may affect anxiety and the perception of disease control in patients treated with natalizumab. A total of 62 relapsing–remitting multiple sclerosis patients were recruited from 2019 to 2020. All patients received conventional infusion treatments with natalizumab, along with a screening protocol for PML. Three clinical assessments were considered: at the beginning of the study (T0), after 3 months (T1) and after 6 months (T2). Patients were classified into three levels of risk, where level 1 represented a low risk of PML and level 3 a high risk. This classification determined treatment and screening protocol, i.e., the frequency of performing the Stratify test and the brain 3T NMR exam, as well as the frequency of infusion treatments. Anxiety and perception of disease control were assessed at T0, T1, and T2 by a skilled psychologist. The Friedman test and the Wilcoxon signed-rank test were used to compare outcomes at baseline with the two follow-ups. Statistical test results showed that the risk of PML (per 1000 patients) was significantly lower in women than in men (W = 198.5; *p* = 0.01). Moreover, significant differences between baseline and the two follow-ups were found, both for anxiety (F(2) = 122.6, *p* < 0.001) and for perception of disease control (F(2) = 123.5, *p* < 0.001). In both cases, there was significant improvement between baseline (T0) and the end of the study (T2) in any risk level (*p* < 0.001). An increase in the number of follow-ups, as well as an increase in instrumental investigations, might have a positive effect on both anxiety and the perception of disease control. However, there are many variables involved in the disease process that have an impact on patients’ psychological well-being. Therefore, further and more extensive studies are necessary to evaluate how, and how much, each variable impacts the disease course.

## 1. Introduction

Multiple sclerosis (MS) is a chronic, predominantly immune-mediated disease of the central nervous system and one of the most common causes of neurological disability in young adults [1]. The incidence and prevalence of MS are increasing in both developed and developing countries [2], and the underlying cause remains uncertain. The etiopathogenesis of MS studies the complicated interactions between genetics, geography, and a common viral infection that may lie behind the disease. It can be difficult to divide multiple sclerosis into various disease phenotypes; thus, recent improvements have been made to current criteria. A combination of clinical, imaging, and laboratory markers may be helpful in predicting the clinical course and optimizing treatment in specific patients, as the prognosis of multiple sclerosis differs significantly among individuals [3].

Current multiple sclerosis phenotypic classifications include: primary progressive, secondary progressive, progressive–relapsing, and relapsing–remitting multiple sclerosis (RRMS) [4]. Although “clinically isolated syndrome” (CIS) is considered a distinct clinical entity, it should also be mentioned. Of all of them, the most common is RRMS. This form occurs in about 85% of cases (at onset) and is characterized by acute episodes of neurological deficits (relapses) followed by partial or total regression of symptoms [5]. However, a residual deficit often persists after a relapse, leading to a gradual increasing of disability over the course of the disease.

New treatments, many of them based on existing drugs, are starting to show promise for the more debilitating progressive form of the disease, which until recently has been largely ignored and forgotten. Among them, natalizumab has been shown to be a highly effective treatment for patients with RRMS [6].

Natalizumab is a monoclonal antibody directed against the alpha chain of the VLA-4 integrin (CD49d). Several randomized clinical trials in patients with RRMS have demonstrated that natalizumab substantially reduces clinical and radiological disease activity [7]. Some evidence suggests that the use of natalizumab may result in lower ongoing disease activity when eligible patients start treatment with lower degrees of physical disability, with improvement in a range of outcomes including cognitive function, fatigue, and health-related quality of life [8]. In the study by Lublin et al. [9], natalizumab reduced the clinical severity of relapses and improved recovery from relapse-induced disability in patients.

Despite the great positive impact of clinical management of the disease, natalizumab is associated with the highest risk of developing progressive multifocal leukoencephalopathy (PML), a rare immune-related condition affecting mainly the central nervous system, which is caused by John Cunningham virus (JCV) and can be fatal or provoke severe disability [10,11]. PML appears to occur as a result of a complex interaction between the host and viral factors, leading to the development of a pathogenic form of JCV that can infect and destroy oligodendrocytes in the central nervous system [12]. Clinical symptoms of PML can vary significantly from patient to patient, and may include weakness, paresthesia, cognitive or behavioral changes, gait dysfunction, speech/language difficulties, visual field defects, or seizure. However, PML may be asymptomatic for many months before clinical presentation with new lesions on MRI, and thus patients should be monitored closely via MRI and undergo lumbar puncture for JCV PCR if there is a suspicion of PML [13].

The risk of PML depends on several factors, such as the presence of antibodies to JC virus in the blood (a sign that the subject has been exposed to the virus that causes PML) and their level, the duration of the treatment with natalizumab, and whether or not the subject was treated with immune-suppressing drugs before starting natalizumab. The benefits and risks of treatment should be discussed individually by the specialist and the patient due to the possibility of developing PML. Patients should also receive regular monitoring throughout the course of treatment, and they and their caregivers should be educated about the early symptoms and signs of PML.

According to the latest practice recommendations regarding the use of natalizumab in treating people with MS [14], patient selection, switch protocols that minimize washout periods, management of MS after discontinuation of natalizumab, treatment-withholding procedures, risk management strategies involving anti-JCV antibody testing, education, and extended-interval dosing (EID) are key strategies to optimize the management of MS patients receiving natalizumab [15,16].

The presence of these risk factors seems to be able to influence the perception of disease control of patients taking this drug, as well as the presence of anxiety [17,18]. In particular, JCV-positive patients may experience progressive feelings of concern about the safety of treatment with natalizumab, leading to higher discontinuation rates than initially reported. In this study, we observe the perception of disease control and the level of anxiety in MS patients taking natalizumab. We hypothesize that by increasing the frequency of neurological and nuclear magnetic resonance (NMR) screening, the patients may perceive the risk of PML differently and experience less anxiety.

## 2. Methods

### 2.1. Study Population and Inclusion Criteria

A total of 62 RRMS patients (13 males and 49 females, mean age 40.0 ± 10.8) were consecutively recruited in this study from 2019 to 2020. This was a representative sample of all outpatients who attended the clinic of MS of IRCCS Centro Neurolesi Bonino-Pulejo in Messina, Italy, with at least one dose of natalizumab administered in our centre. Furthermore, only patients with a confirmed diagnosis of MS according to McDonald’s criteria [16] who were cognitively able to answer the study questionnaires and who had signed informed consent were eligible for the study.

### 2.2. Study Design and Setting

**Figure 1:**
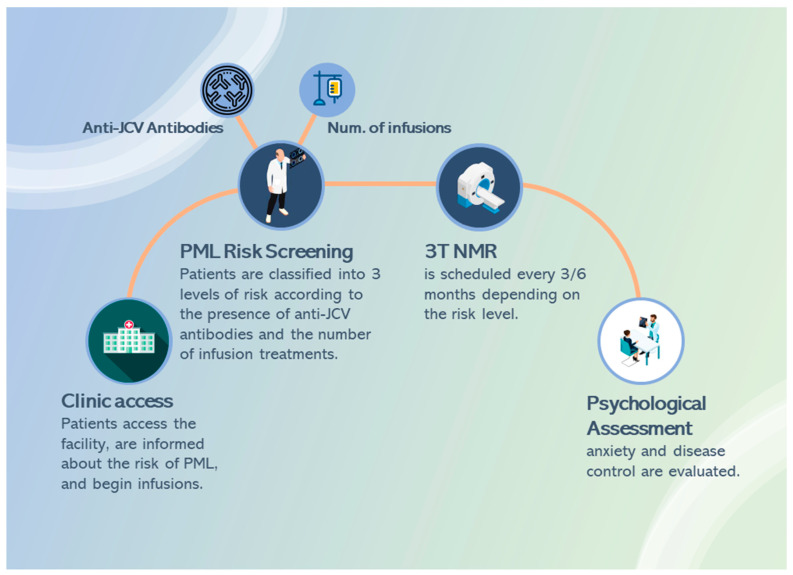
Pathway of MS outpatients treated with natalizumab.

The study was designed as observational. Specifically, it is a longitudinal study with retrospectively collected data. All patients underwent conventional infusion treatments with natalizumab, together with a screening protocol for PML based on European Medicines Agency (EMA) recommendations [19]. According to best practices, before starting treatment with natalizumab, patients and their carers should be informed about the risk of PML (Figure 1).

In addition, patients should be instructed to consult their physician if they think the disease is getting worse, or if they notice new or unusual symptoms. During treatment with natalizumab, patients should be monitored at regular intervals for signs and symptoms of new neurological dysfunction, and a full brain MRI should be performed at least once a year for the duration of treatment. For patients at higher risk of PML, more frequent MRIs (e.g., every 3–6 months) using an abbreviated protocol (e.g., FLAIR, T2-weighted and DW imaging) should be considered, as early detection of PML in asymptomatic patients is associated with improved PML outcomes.

Three clinical evaluations were considered: at the beginning of treatment (T0), after 3 months (T1), and after 6 months (T2).

### 2.3. Assessment of Risk of PML

In accordance with EMA recommendations [20], specialized neurologists identified and assessed each individual’s risk of PML according to current knowledge of natalizumab-associated PML (the presence of anti-JCV antibodies and the number of infusion treatments received over 2 years) [21,22,23,24]. Thereafter, brain NMR screening was scheduled once a year for patients with low risk, every 6 months for patients with medium risk, and every 3 months for patients with high risk [20]. The risk of PML was monitored using a checklist administered before infusion, to assess whether the risk level had changed during treatment, and through the Stratify test [25].

All patients were classified into 3 levels of risk according to two risk factors, where level 1 represents a low risk of PML (when no risk factors were detected), level 2 a medium risk (at least one risk factor), and level 3 a high risk (presence of both risk factors). This classification determined the treatment and screening protocol, i.e., the frequency of performing the brain 3T NMR examination, as well as the frequency of infusion treatments (Table 1).

### 2.4. Assessment of Anxiety and Perception of Disease Control

The Hamilton Anxiety Rating Scale (HAM-A) and an ad hoc 0–10 Likert scale were administered blindly by a skilled psychologist to evaluate the patient’s level of clinical monitoring anxiety and perception of disease control, respectively. The HAM-A consists of 14 items, each defined by one or more symptoms, and evaluates both the psychological aspects (such as mood, emotions, and mental agitation) and the physical symptoms of anxiety. Each item is scored on a scale from 0 (not present) to 4 (severe), with a total score range of 0–56, where <17 indicates mild severity of anxiety, 18–24 from mild to moderate severity, and 25–30 from moderate to severe [26]. Concerning the perception of disease control, an affirmative opinion about the issue was administered to the participants, asking them to express their degree of agreement from 0 to 10, where 10 indicates greater perception of disease control.

### 2.5. Statistical Analysis

Statistical analysis was carried out using the 3.5.0 version of the open-source software R (R Core Team, Vienna, Austria), considering α = 0.05 as the level of significance. A nonparametric one-way repeated measures analysis, to assess the differences between the three assessments in each risk level group, was performed using the Friedman test (stats package), since the results of the Shapiro–Wilk test indicated a non-normal distribution of the target variables (stats). For each outcome, the Levene’s test (lawstat) was used to assess the equality of variances at different times. For variables where the Friedman’s test reached significance, the Wilcoxon signed-rank test (stats) was used to perform within-group pairwise comparisons, considering Bonferroni’s correction (post hoc analysis). Continuous data were expressed as mean ± standard deviation or as median ± first-third quartile, as appropriate, whereas categorical data were expressed as frequencies and percentages. The Kruskal–Wallis test (stats) was used to assess baseline differences across the groups with different risks, whereas the Wilcoxon rank-sum test (stats) was used to compare the outcomes between men and women. Spearman’s rank correlation coefficient was used to assess the correlations between T2–T0 changes (e.g., subtraction between the score at the end of the study and the baseline) of each outcome variable and PML risk rate (per 1000 patients). A mixed-effects analysis (nlme, lme4, and lmerTest) was also performed in order to investigate the relationship between outcome measures changes, risk level (L1, L2, L3), and assessment time (T0, T1, T2). In fact, these factor variables were considered fixed effects, while the subject’s variability was considered a random effect. Models included intercepts and slopes for the effect of level of risk. The interaction between the fixed effects was also considered if it added information to the model according to the AIC criteria. An ANOVA (stats) has been used to compare the full model (including the effect ‘risk level’) against the model without this effect, whereas an ANOVA for repeated measures has to be included in the mixed-effects analysis.

## 3. Results

### 3.1. Descriptive Analysis

The study population included mainly women (about 79%), with a mean EDSS of 4.2 ± 1.1, a mean disease duration of 9.1 ± 2.8 years, and a mean duration of treatment with natalizumab of 6.6 ± 3.2 years. A description of the sample’s characteristics is reported in Table 2. Almost half of the sample had a medium–high risk of PML (41.9%). No significant difference in age was found between women and men (W = 329; *p* = 0.67), or among patients in different levels of risk classification (KW (2) = 0.803; *p* = 0.80). The risk of PML (per 1000 patients) was significantly lower in women than in men (W = 198.5; *p* = 0.01) and increased significantly according to risk levels (KW (2) = 28.67; *p* < 0.001).

### 3.2. Anxiety and Perception of Disease Control

The Friedman test and Wilcoxon signed-rank test showed significant differences from baseline to the two follow-ups for both anxiety (F(2) = 122.6, *p* < 0.001) and perceived disease control (F(2) = 123.5, *p* < 0.001). As reported in Table 3, we found significant improvements in anxiety and perception of disease control between baseline (T0) and the end of the study (T2) at each risk level (*p* < 0.001). Post hoc analysis showed significant differences between each pair of assessment times.

The risk of PML (per 1000 patients) was found to not be correlated with T2–T0 changes in HAM-A scores (r = −0.08; *p* = 0.52) or with T2–T0 changes in perceived disease control scores (r = 0.14; *p* = 0.28). In fact, the results of ANOVA analysis showed a significant difference in assessment time both for HAM-A (F(1) = 444.66; *p* < 0.001) and perceived disease control (F(1) = 645.91; *p* < 0.001), but not in risk level both for HAM-A (F(1) = 1.97; *p* = 0.162) and perceived disease control (F(1) = 2.22; *p* = 0.138), as reported in Table 4.

**Figure 2:**
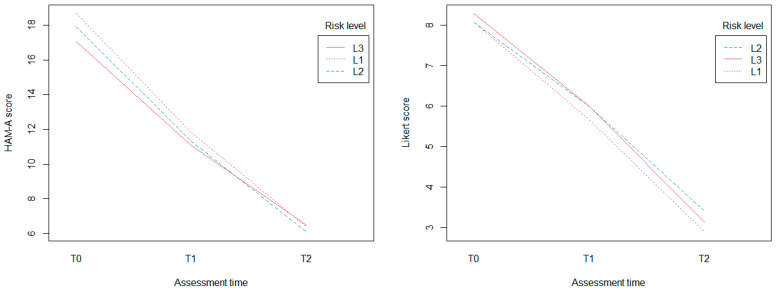
Interaction plot showing how the relationship between risk level and outcome measures depends on the value of assessment time. HAM-A = Hamilton Anxiety Rating Scale.

The results of the mixed-effects analysis also showed that risk classification has no effect on both anxiety (X^2^(6) = 3.65; *p* = 0.72) and disease control (X^2^(6) = 3.75; *p* = 0.71). However, a significant trend in HAM-A mean scores about the class of risk L3 (t = 1.71; 0.06) was found, as shown in Figure 2.

## 4. Discussion

Patients with RRMS benefit greatly from the use of natalizumab as a therapeutic option, as it has demonstrated exceptional efficacy in reducing clinical relapse, preventing new MR activity, and delaying the progression of functional disability. In addition, natalizumab has also been shown to have positive effects on balance and motor symptoms, cognition, depression, and fatigue, which are important disabling symptoms of MS that mostly reduce quality of life [6].

Natalizumab is considered a first-line treatment option for RRMS, but there is a chance of adverse events that could cause PML [27]. The recent guidelines on the use of natalizumab and numerous safety campaigns for MS patients have not changed the incidence of PML from natalizumab, and patients’ lack of confidence in the drug is growing [20]. The risk of PML is the most common reason for discontinuation of treatment with natalizumab. Currently, it is possible to make a more calculated risk assessment for individual patients depending on their JCV antibody status, previous immunosuppressant use, and treatment duration; this may imply a change in risk acceptance for individual patients [28]. In addition, so far, it is known that patient selection, switching protocols that minimize washout periods, proper management of MS after discontinuation of natalizumab, treatment-withholding procedures, education, and extended-interval dosing are key strategies for optimizing the management of MS in patients receiving natalizumab [29]. An extremely important element linked to the discontinuation of the drug is the risk–benefit profile. The aspect of switching from one drug to another must be managed carefully in consideration of the possible risk of adverse events and, at the same time, the possible reactivation of the inflammatory activity which is typical when switching from high-activity drugs to those with low efficacy. Based on this consideration, treatment with natalizumab continues until the risk outweighs the clinical benefit for the patient. In this study, we evaluated attitudes towards natalizumab in patients with MS in association with PML risk. Best practices that prescribe frequent clinical visits seem to provide patients with reassurance and positively influence their perception of disease control, highlighting the importance of integrating chronic disease management into patients’ daily lives to enhance adherence to medical treatments [30]. Indeed, the results showed significant differences in the level of anxiety scores between baseline and the subsequent follow-ups. In addition, the perception of being able to exert active control over the disease increased. Cognitive representations or ideas regarding the disease are crucial indicators of how patients will act when ill, and are related to a range of positive health outcomes [31]. Patient-perceived severity can be considered a proxy for beliefs about the objective controllability of disease, and is related to health behavior under certain conditions such as perceived vulnerability [32]. According to data from the literature [33], better disease perception is often associated with better disease management and better health outcomes. Therefore, it is becoming increasingly crucial to consider how to help patients have more favorable impressions of their illnesses. Moreover, patients’ perceptions of their illnesses can be influenced by the receipt of adequate and personalized health information. As a result, it is appropriate to ask whether there are relationships between health information behavior and disease perceptions and, if so, what aspects of that behavior are related to the more optimistic view of the disease that we want patients to develop. In MS patients taking natalizumab, this represents an important factor in improving treatment adherence and disease management. In addition, improved disease perception empowers patients to actively participate in their care decisions, fostering better communication with healthcare professionals and, ultimately, enhancing treatment efficacy.

This study emphasizes the importance of psychological variables within the care pathway. A chronic illness changes a person’s sense of self, as it replaces their previous healthy identity with an illness identity. Restoring a sense of self involves an adaptive, ongoing process. Increased anxiety in MS is linked with poor disease management, worse medication adherence, and worse perception of the intrusiveness of the condition. In addition, anxiety is related to a higher number of relapses, shorter time to relapses, and predicts disease reactivation [34].

A simple change in a patient’s course of care, treatment, or rehabilitation could have an important effect on their psychological outcomes. It is possible to assume that an increase in the number of follow-ups could help patients to feel more confident about their disease, greatly improving their quality of life. However, variables involved in the disease process that could influence a patient’s psychological aspects are copious. Therefore, future studies should focus on longitudinal research to assess the long-term effects of anxiety reduction and disease perception improvement on patient outcomes, including disease progression and QoL. Future work should also continue with the development of new biomarkers that may better predict an individual’s risk of PML and further improve natalizumab’s safety.

## 5. Limitations

This work presents some significant methodological limitations. First, the small sample size in each group and the retrospective study design do not allow the generalization of the results obtained. In addition, the three classes of risk level included a different number of subjects. Employing a balanced case-control study with a control group might have allowed for more definitive conclusions. Second, we have no information on how well patients took in the information provided about the risk of PML. Third, the perception of disease control was evaluated using a unidimensional Likert scale instead of a validated tool. Patient behavior can be influenced by the physician’s attitude; we did not take this into account in this study. Finally, it is not unlikely that the severity of the disease may have affected the willingness to take a risk.

## 6. Conclusions

Because of the increased risk of developing PML, the benefits and risks of treatment should be reconsidered individually by the specialist and the patient; patients should be monitored at regular intervals throughout the duration of treatment, and they and their caregivers should be educated about the early signs and symptoms of PML. Furthermore, ongoing research is exploring biomarkers for identifying patients at risk of developing PML, while recent advancements in MRI usage aim to detect PML in its early stages. However, a very important aspect is the recognition of psychological constructs, such as mood alterations or disease perception of chronic diseases like MS, which are fundamental to providing patient-centered care and achieving better therapeutic outcomes. Indeed, JCV-positive patients could experience progressive feelings of concern regarding the safety of treatment with natalizumab, which could be amplified by symptoms of anxiety. These patients could be less confident in their decision to continue treatment, feel less confident overall, and be more fearful of PML. In our clinical experience, even JCV-positive patients who are fully aware of the high personal risk of developing PML are willing to continue treatment with natalizumab. A possible explanation for the discrepancy between anxiety and safety could be that patients feel more comfortable with the rigorous follow-up schedule that we adopt. This cure model allows for greater control of variables such as anxiety, which can have negative repercussions in the treatment process of subjects with MS.

## Figures and Tables

**Table 1:** Risk classification, treatment, and associated screening protocols. JCV is the presence/absence (pos/neg) of anti-JCV antibodies, and N. infusions indicates the treatment duration (less or more than 24 months).

Risk Levels	JCV		N. Infusions		Time Interval	
	Neg	pos	≤24	>24	3T NMR	Infusions
Level 1	•				1 year	4 weeks
Level 2		•	•		6 months	4 weeks
Level 3		•		•	3 months	6 weeks

**Table 2:** Study’s population description according to risk classification of PML.

	Risk Classification		
	Level 1	Level 2	Level 3
Participants, *n* (%)	36 (58.1)	12 (19.3)	14 (22.6)
Males, *n* (%)	5 (38.4)	5 (38.5)	3 (23.1)
Age (years), mean ± SD	43.5 ± 10.5	45.7 ± 8.4	42.1 ± 13.4
Risk rate, mean ± SD	0.8 ± 2.4	2.7 ± 3.4	5.9 ± 4.0

Legend: SD = standard deviation.

**Table 3:** Friedman’s test results and significant differences detected by the Wilcoxon signed-rank test with Bonferroni’s correction.

	Scores at Each Examination (Median [First–Third Quartile])			One-Way Repeated Measures Analysis		Post Hoc Analysis	
	T0	T1	T2	Test (df)	*p*-Value	Significant Differences	*p*-Value
HAM-A Risk L1	19.5 [17.0–21.0]	11.0 [10.0–14.0]	6.5 [5.0–8.0]	71.5 (2)	**<0.001**	T1–T0	**<0.001**
						T2–T0	**<0.001**
						T2–T1	**<0.001**
HAM-A Risk L2	14.5 [14.0–15.7]	18.0 [9.7–12.2]	6.0 [5.7–6.2]	23.5 (2)	**<0.001**	T1–T0	**0.003**
						T2–T0	**0.002**
						T2–T1	**0.002**
HAM-A Risk L3	17.0 [15.2–18.7]	10.0 [10.0–13.2]	6.0 [5.2–8.0]	27.5 (2)	**<0.001**	T1–T0	**0.002**
						T2–T0	**0.001**
						T2–T1	**0.001**
pdc Risk L1	3.0 [2.7–4.0]	6.0 [5.0–6.0]	8.0 [7.0–9.0]	72.0 (2)	**<0.001**	T1–T0	**<0.001**
						T2–T0	**<0.001**
						T2–T1	**<0.001**
pcd Risk L2	3.5 [3.0–4.0]	5.5 [5.0–7.2]	8.0 [7.7–8.2]	24.0 (2)	**<0.001**	T1–T0	**0.002**
						T2–T0	**0.002**
						T2–T1	**0.002**
pcd Risk L3	3.0 [3.0–4.0]	6.0 [6.0–6.0]	8.0 [7.2–9.0]	27.5 (2)	**<0.001**	T1–T0	**<0.001**
						T2–T0	**<0.001**
						T2–T1	**0.001**

Legend: HAM-A = Hamilton Anxiety Rating Scale; pcd = perceived disease control; Test (df) = statistical test value (degree of freedom); T0 = baseline; T1 = 3 months follow-up; T2 = end of the study. Significant results are in bold.

**Table 4:** ANOVA analysis with results of repeated measures.

		Sum Sq	Mean Sq	Test (df)	*p*-Value
HAM-A	Risk level	19	19	1.97 (1)	0.162
	Assessment time	4321	4321	444.66 (1)	**<0.001**
	Risk–time	14	14	1.43 (1)	0.233
	Residuals	1769	10	(182)	-
Perceived disease control	Risk level	2.7	2.7	2.22 (1)	0.138
	Assessment time	800.2	800.2	645.91 (1)	**<0.001**
	Residuals	226.7	1.2	(183)	-

Legend: HAM-A = Hamilton Anxiety Rating Scale; Risk–time = interaction term risk for time; Test (df) = statistical test value (degree of freedom). Significant results are in bold.

## Data Availability

Data are contained within the article.
